# Person‐centred care and short oral treatment for rifampicin‐resistant tuberculosis improve retention in care in Kandahar, Afghanistan

**DOI:** 10.1111/tmi.13716

**Published:** 2022-01-17

**Authors:** Anita Mesic, Sadiqullah Ishaq, Waliullah H. Khan, Atiqullah Mureed, Htay Thet Mar, Ei Ei Khaing, Elkin Bermudez‐Aza, Letitia Rose, Lutgarde Lynen, Mohammad Khaled Seddiq, Hashim Khan Amirzada, Kees Keus, Tom Decroo

**Affiliations:** ^1^ Médecins Sans Frontières Amsterdam The Netherlands; ^2^ Institute of Tropical Medicine Antwerp Antwerp Belgium; ^3^ Médecins Sans Frontières Kabul Afghanistan; ^4^ Médecins Sans Frontières Kandahar Afghanistan; ^5^ National Tuberculosis Control Programme Ministry of Public Health Kabul Afghanistan; ^6^ Research Foundation Flanders Brussels Belgium

**Keywords:** Afghanistan, drug‐resistant tuberculosis, Kandahar, person‐centred care, short oral regimen

## Abstract

**Objectives:**

To describe the effect of adaptations to a person‐centred care with short oral regimens on retention in care for rifampicin‐resistant TB (RR‐TB) in Kandahar province, Afghanistan.

**Methods:**

The study included people with RR‐TB registered in the programme between 01 October 2016 and 18 April 2021. From 19 November 2019, the programme implemented a trial investigating the safety and effectiveness of short oral RR‐TB regimens. During the trial, person‐centred care was adapted. We included the data from people living with RR‐TB treated in the period before and after the care model was adapted and applied Kaplan‐Meier statistics to compare rates of retention in care.

**Results:**

Of 236 patients registered in the RR‐TB programme, 146 (61.9%) were registered before and 90 (38.1%) after the model of care was adapted. Before adaptations enhancing person‐centred care, pre‐treatment attrition was 23.3% (*n* = 34/146), whilst under the adapted care model it was 5.6% (*n* = 5/90). Attrition on treatment was 22.3% (*n* = 25/112) before adaptations, whilst during the study period none of the participants were lost‐to‐follow‐up on treatment and 3.3% died (*n* = 3/90).

**Conclusions:**

As person‐centred care delivery and treatment regimens were adapted to better fit‐specific contextual challenges and the needs of the target population, retention in care improved amongst people with RR‐TB in Kandahar, Afghanistan.

## INTRODUCTION

Management of rifampicin‐resistant tuberculosis (RR‐TB) remains one of the main obstacles in reaching the goal of TB elimination by 2035 [1]. There are multiple reasons for unfavourable outcomes, including suboptimal, long or toxic treatment regimens, but also a ‘one‐size‐fits‐all’ approach to treatment delivery. In 2016, WHO recommended a second‐line injectable‐containing short regimen for treating multidrug‐resistant TB (MDR‐TB) [2]. In 2018, building on lessons learnt from experiences with the new and repurposed antituberculosis drugs (such as bedaquiline, delamanid, linezolid and clofazimine), all‐oral regimens were recommended [3, 4, 5]. As the performance of regimens also depends on local factors, countries are encouraged to pilot short oral regimens under operational research conditions [6].

Acknowledging that people living with TB face multiple challenges in addition to TB disease [7] and that better access to new diagnostic or treatment tools alone cannot improve RR‐TB care outcomes, person‐centred care has been introduced as one of the pillars of the EndTB strategy, which calls for integration of health services, inclusion of social support as part of clinical care, and improved collaboration between clinical providers and community and civil society stakeholders [8]. Whilst person‐centred care is widely embraced, it is still vague what this approach entails. There is limited evidence on how to operationalise RR‐TB person‐centred care in programmatic contexts. In a perspective paper, Odone et al. present what could be four essential components of TB patient‐centred care. First, a holistic approach should be used, whereby patients are defined by more than just their physical illness. Also, the social, cultural and economic reality of people, their families and communities should be considered. Second, the care package should be individualised, thus considering, and responding to, the needs of individual persons. Third, people living with TB should be empowered to take an active role in their own follow‐up. Finally, decision‐making should involve people when it comes to making decisions about their own health and care [3].

Since 2016, in collaboration with the National Tuberculosis Control Programme (NTP), Médecins Sans Frontières (MSF) has been providing RR‐TB care in Kandahar, Afghanistan [9]. Afghanistan is affected by a protracted conflict. Access to humanitarian medical services is limited [10] and TB is a public health crisis that disproportionally affects women and children [11]. Despite enormous efforts of the NTP to decentralise RR‐TB care, multiple barriers persist, including distances between communities and RR‐TB clinics, limited access to quality‐assured laboratory services, limited qualified health staff, insecurity, economical and cultural barriers, and TB‐related stigma [12].

Recently, we reported that RR‐TB can be successfully treated in a complex setting like Kandahar but might still be affected by high attrition rates [9]. We concluded that our programme needed to adopt strategies to improve outcomes along with the cascade of care and increase the acceptability of treatment delivery. We also addressed problems that were not purely physical as we strengthened psychosocial services, including counselling, and social and peer support to improve psychological and cognitive well‐being of people receiving RR‐TB care in our programme. To address individual health needs, we integrated care for the most common co‐morbidities (diabetes, mental health) to be provided all at the same venue. We provided material support (transport allowance and food) to all, plus accommodation in Kandahar for those who lived far away. Given the local cultural, social and economic context, long regimens were very challenging for patients. We therefore piloted regimens with shorter treatment duration. Participants and their families were empowered to actively participate in their own care. Persons with RR‐TB were informed about the features of self‐administered treatment (SAT) and directly observed treatment (DOT) and were involved in the choice between both approaches. Those who chose SAT and who demonstrated a good understanding of the importance of adherence and treatment intake, received monthly drug‐supplies. TB does not only affect individuals but also their families and communities. Therefore, our model embraced a family approach. Families were supported to improve the acceptability and uptake of care delivery, and thus increase TB detection and adherence to care. The main objective of the study was to describe the model of care, baseline characteristics and early outcomes of our cohort. We also compare retention in care before and after the additional measures were implemented.

## METHODS

### Study population

The study includes participants with RR‐TB enrolled in the MSF programme in the period from 01 October 2016 to 18 April 2021. The study period is divided in the period before (01 October 2016–18 November 2019) and after (19 November 2019–18 April 2021) the implementation of the person‐centred care. In the period after the adaptation of the model of care, a clinical trial ‘Effectiveness and tolerability of short treatment regimens for drug‐resistant tuberculosis treatment in Kandahar, Afghanistan’ was implemented in the MSF programme [3]. With this study we describe person‐centred care implemented in the programme and we present baseline characteristics of the study population and proportion of those retained in care before and after the implementation of measures enhancing person‐centeredness.

### Comprehensive and person‐centred RR‐TB care model

Throughout the study period, people were referred with presumptive diagnosis of RR‐TB to the MSF programme. Each person was then diagnosed with confirmed RR‐TB based on Xpert MTB/RIF result or with clinical RR‐TB diagnosis based on their contact history and clinical presentation. How RR‐TB care was provided before measures enhancing person‐centeredness model was described previously [13].

**FIGURE 1:**
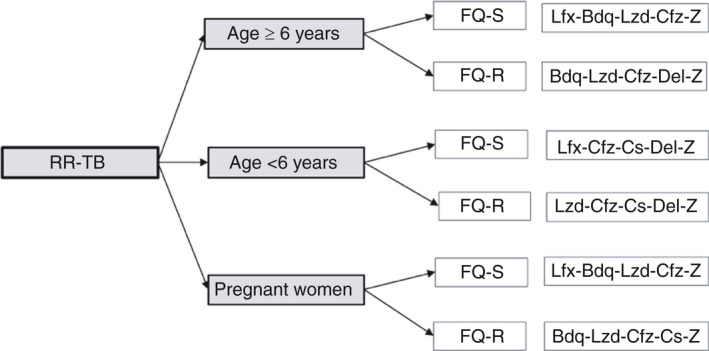
Short oral treatment regimens used in Kandahar cohort. RR‐TB, rifampicin‐resistant tuberculosis; FQ‐S, fluoroquinolone susceptible; FQ‐R, fluoroquinolone resistant; Bdq, bedaquiline; Lfx, levofloxacin; Cfz, clofazimine; Lzd, linezolid; Z, pyrazinamide; Del, delamanid; Cs, cycloserine

Measures enhancing person‐centredness were implemented stepwise. As a first step, those registered in the RR‐TB care programme were offered participation in the trial on short oral regimens. Six short (9–12 months) standardised all‐oral treatment regimens (Figure 1) were available. Their composition followed the WHO recommendations [3] and differed considering age, fluoroquinolone susceptibility and pregnancy (Figure 1). Those not eligible for the trial (previous exposure to second‐line antituberculosis medications, contraindications for any of the medications in the regimen, severe clinical condition that requires individualised approach) or not willing to provide consent for participation, were treated by individualised regimen based on WHO guidelines [15].

After the first month of treatment, participants could choose between continuing DOT and SAT. Treatment was ambulatory unless the participants' clinical condition required hospitalisation.

During the first month of treatment, participants stayed in Kandahar, and with their family members received health and therapeutic education, adherence counselling and psychosocial support. Health education focused on infection prevention and control techniques. Therapeutic education included a review of prescribed drugs, their dosing, and early recognition and management of adverse events. Adherence counselling addressed the importance of adherence and how pill‐intake could be incorporated into daily life. Psychosocial support explored adherence barriers, how these could be overcome, and an individualised support plan was designed.

**FIGURE 2:**
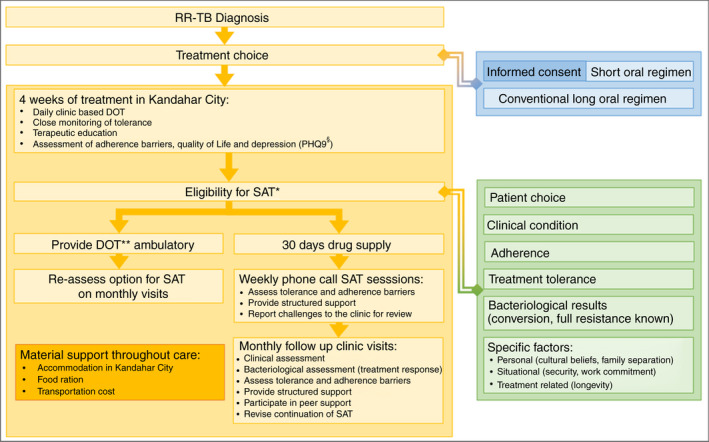
Patient‐centred model of care for RR‐TB in Kandahar, Afghanistan. *Self‐administered treatment; **Directly observed treatment

During the first 2 weeks, treatment was delivered as clinic‐based DOT, followed by gradual training and preparation for SAT. For participants younger than 15 years, a caretaker responsible for treatment administration was identified. Before the end of the first treatment month, the participant's eligibility for SAT was assessed (Figure 2) by the interdisciplinary medical team consisting of medical doctors, nurses, counsellors and health promotion staff. They considered the patient's clinical condition, treatment tolerance, knowledge about RR‐TB and their treatment regimen, demonstrated adherence and persisting barriers. Monthly follow‐up visits were scheduled. Counsellors contacted participants who provided phone numbers, weekly, to assess treatment tolerability, adherence barriers and to provide counselling. If required, they were referred to the clinic. If participants came from an accessible and secure area, community workers tried to reach their homes to provide individualised support each week. During monthly follow‐up visits at the clinic, SAT eligibility was reassessed considering the same parameters used at baseline. In case of suboptimal adherence, the team enhanced the support plan, including additional intensive counselling sessions, active participation in patient groups, peer‐provided counselling and, if feasible, home visits. Throughout the treatment, all participants received monthly food parcels and hygiene packages, their travel costs were reimbursed and, if needed, MSF supported their accommodation in Kandahar.

### Contact tracing

Contact tracing was implemented at the start of the programme and was strengthened as part of the person‐centred model of care. This included symptom screening of all adults (age >15 years) household members either during the clinic visit or by home visit when feasible and during the first month of the index person registration. Those with TB symptoms underwent clinical, radiographical and bacteriological (Xpert MTB/RIF) testing. For household members 15 years old or younger, a clinical examination and radiological and bacteriological testing was performed, regardless of their symptoms. All contacts were reassessed every 3 months during the period that that index person was treated, including the 12‐month post‐treatment follow‐up.

### Data collection

Data were collected from standardised patient forms and encoded in the MSF TB‐programme electronic database (Koch6) and in a secure web‐based software platform designed to support data capture for research studies (Research Electronic Data Capture). Data retrieved from the MSF TB‐programme database were exported into statistical software STATA (version 14.2) for analysis.

### Data analysis

Categorical variables were summarised using frequencies and percentages. When distribution of continuous variables was not normal, we calculated medians and interquartile range (IQR). The Wilcoxon rank‐sum test assessed whether medians between groups were statistically different. Odds ratios with 95% confidence intervals were calculated to assess associations between demographical and clinical characteristics and the study period. Kaplan‐Meier curves were plotted to assess differences in retention before and after the implementation of the person‐centred model of care. Observation time was defined as the time between the date of registration into the programme and the date of the outcome (death or lost‐to‐follow‐up) or at the end of the study period, whichever occurred first. For participants who did not start the treatment but were registered into the cohort, we did not have dates for pre‐treatment lost‐to‐follow‐up or death. Therefore, we used a proxy date, which was 7 days from registration, based on the average time for treatment initiation in the programme. The log‐rank test was used to determine if the difference between survival curves were significant. As previous use of second‐line drugs was an exclusion criterion for the novel short regimens, we performed a sensitivity analysis for which we excluded those who were previously exposed to second‐line antituberculosis drugs (*n* = 3) also from the historical cohort of participants who were registered in the programme before the start of the measures enhancing person‐centred care.

### Ethical approval

The clinical trial was approved by the Institutional Review Board (IRB) of the Ministry of Public Health, Afghanistan (A08190063); the IRB of the Institute of Tropical Medicine, Antwerp (1329/19); the Ethical Committee, University of Antwerp (19/49/566); and the MSF independent Ethical Review Board (ERB; 1933/19). All participants 18 years or older provided written informed consent to participate in the study. For those younger than 18 years old, informed consent was signed by the guardian with assent given by children between 15 and 18 years old. Retrospective analysis that included participants registered in care outside of the clinical trial was approved by the IRB, Ministry of Public Health, Afghanistan (A07190052), and fulfilled the exemption criteria set by the MSF independent ERB [23] for *a posteriori* analyses of routinely collected clinical data, which requires patients in MSF programmes to have given consent for secondary use of their data at the time of treatment.

## RESULTS

### Baseline demographical and clinical characteristics

**FIGURE 3:**
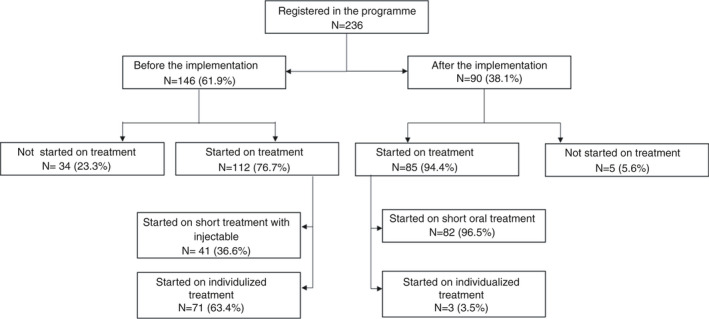
Flow of the participants registered in the programme between 01 October 2016 and 18 April 2021 (*n* = 236)

There were 236 patients registered in the RR‐TB programme: 146 (61.9%) before and 90 (38.1%) after the implementation of the person‐centred model of care (Figure 3). Before and after the implementation 112 (76.7%) of 146 and 85 (94.4%) of 90 participants registered with the diagnosis of RR‐TB started on treatment, respectively. In the period before the implementation, of 34 (23.3%) not retained in care between diagnosis and treatment start, 10 (29.4%) died and 24 (70.6%) were lost‐to‐follow‐up (refused treatment). During the period after implementation of the person‐centred model, three patients died and two were lost‐to‐follow‐up before starting treatment. Of the remaining 85 patients, 82 (96.5%) were eligible and consented to participate in the study and chose the short oral regimen instead of the WHO long all‐oral treatment regimen. Non‐eligibility for the study was due to previous exposure to second‐line anti‐TB drugs (*n* = 2) and severe RR‐TB meningitis (*n* = 1). Non‐eligible patients were started on individualised treatment regimens according to the national guidelines [11] and are not included in this analysis.

Table 1 shows demographical and clinical characteristics of the participants registered in the programme before and after implementation of the person‐centred model of care. Median age at treatment start was 30.1 years (IQR 22.6, 45.0) and 25.1 years (IQR 14.2, 40.0) before and after the implementation, respectively (*p* = 0.09). Participants registered in the programme after the implementation of person‐centred model of care were more likely to be ≤15 years old (OR 2.85, 95% CI 1.37–5.88, *p* = 0.003) and more likely to be diagnosed through contact tracing activity (OR 3.09. 95% CI 1.65–5.75, *p* = 0.0002). In the period after the implementation of the person‐centred model of care participants were higher odds of not having bacteriological confirmation of disease with 8.3 (95% CI 4.00–16.67, *p* < 0.001) and 5.88 (95% CI 2.94–12.5, *p* < 0.001) odds ratio of negative baseline smear microscopy or culture, respectively, when compared with participants registered before the model of care was adapted.

**TABLE 1:** Comparison of the baseline demographical and clinical characteristics of patients enrolled in the study after and before the enhancement of the person‐centred model of care (*n* = 236)

Characteristic	Category	Before the implementation *N* = 146		After the implementation *N* = 90		Odds ratio	95% CI	*p*‐value
		*N*	%	*N*	%			
Gender	Male	58	63.0	34	37.0	1	0.63–1.86	0.77
	Female	88	61.1	56	38.9	1.08		
Age group (years)	>15	130	66.0	67	34.0	1	1.37–5.88	0.003
	≤15	16	41.0	23	59.0	2.85		
Province	Kandahar	56	72.3	21	27.3	1	1.12–3.73	0.02
	Outside of Kandahar	90	56.6	69	43.4	2.04		
Contact	No	121	68.8	55	31.2	1	1.65–5.75	0.0002
	Yes	25	41.7	35	58.3	3.09		
Site of TB infection	Pulmonary	143	63.3	83	36.7	1	1.00–1.04	0.03
	Extrapulmonary	3	30.0	7	70.0	4.00		
Body Mass Index (kg/m^2^)	<18.5	44	62.9	26	37.1	1	0.73–2.42	0.89
	≥18.5	75	56.0	59	44.0	1.33		
	Data unavailable	27	18.5	5	5.5			
Chest radiography^a^	Non‐severe	46	64.8	25	35.2	1	0.67–2.14	0.55
	Severe	100	60.6	65	39.4	1.20		
Baseline smear	Positive	110	82.1	24	17.9	1	4.00–16.67	<0.001
	Negative	33	36.3	58	63.7	8.3		
	Data unavailable	9	2.1	8	8.9			
Baseline culture^b^	Positive	89	82.4	19	17.6	1	2.94–12.5	<0.001
	Negative	40	44.0	51	56.0	5.88		
	Data unavailable	17	11.6	20	22.2			
Treatment regimen^c^	Short with second‐line injectable	41	28.1	–	–			
	Individualised	71	48.6	3	3.3			
	Short oral	–	–	82	91.1			
	Not initiated	34	23.3	5	5.6			

^a^ Severe radiographic result includes (unilateral or bilateral) cavities and/or other bilateral pathological findings.

^b^ During the COVID‐19 pandemic and the national lockdown, transportation of samples was interrupted; frozen samples were sent to the supranational laboratory in 2021 and at the time of the study, results were still pending.

^c^ Short standardised regimen (9–11 months) for multidrug‐resistant TB consisting of: amikacin/kanamycin, moxifloxacin, ethionamide, high dose isoniazid, clofazimine and pyrazinamide, as recommended by the WHO in 2016 [6]; Individualised regimen consisting of at least four effective drugs based on bacteriological findings, tolerance and previous treatment history. Regimen is provided for 18–24 months; short oral regimen as illustrated in Figure 2.

### Treatment delivery

At the time of analysis, 10 (11.7%) participants were in their first month of treatment and 75 (88.2%) participants were eligible for SAT assessment (Table 2). Amongst those eligible for SAT assessment four (5.3%) were hospitalised based on clinical condition. All other participants (*n* = 71) chose to receive their treatment at home through SAT. The median time between treatment start and the start of SAT was 31 days (IQR 2,346). In 14 participants SAT was interrupted, most frequently because of hospitalisation indicated by their clinical status (*n* = 7, 50%). The median time to SAT interruption was 19 days (IQR 16,145). In two participants SAT was interrupted due to missed appointments. Altogether, half (*n* = 7) of 14 participants restarted SAT after the interruption, including two participants with adherence barriers. Those who did not restart SAT at the time of analysis were either hospitalised (*n* = 4) or preferred to receive their treatment by DOT (*n* = 3).

**TABLE 2:** Self‐administered treatment outcomes (*n* = 75)

Characteristic	Category	*N*	%
Started on SAT^a^	Yes	71	94.6
Caretaker available amongst those on SAT	Yes	60	88.2
Relation to the caretaker	Child	6	10.0
	Parent	20	33.3
	Sister/Brother	3	5.0
	Spouse	19	31.7
	Other	12	20.0
Access to phone amongst those on SAT	Yes	60	84.5
Access to home amongst those on SAT	Yes	21	33.8
Other family members with DR‐TB amongst those on SAT	Yes	31	45.6
SAT interrupted	Yes	14	20.6
Reason to interrupt SAT	Clinical condition	7	50.0
	Non‐adherence	2	14.3
	Other^b^	5	35.7
Restarted SAT after interruption	Yes	7	50.0
Time to start SAT	Median (days)	31	IQR 22,46
Duration of SAT before interruption	Median (days)	19	IQR 16,145

^a^ At the time of analysis there were *n* = 10 participants in their first month of treatment. Here data is shown for those beyond one month and eligible to start SAT (*n* = 75).

^b^ Other = Preferable stay in Kandahar due to security issues and preferable choice to receive treatment by DOT (*n* = 4); Hospitalisation of the mother (*n* = 1).

### Treatment outcomes

**FIGURE 4:**
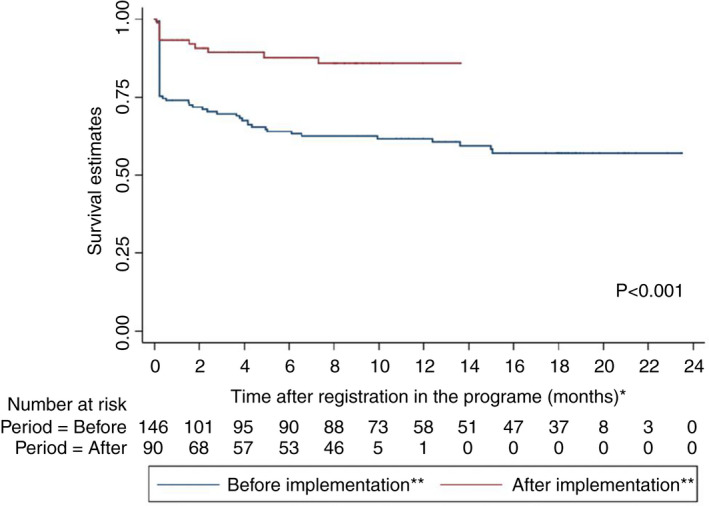
Kaplan‐Meier survival estimates of retention in care stratified by the registration period. *Observation time was defined as the time between the date of registration into the programme and the date of the outcome (attrition) or at the end of the study period whichever occurred the first. For participants who did not start the RR‐TB treatment but were registered we did not have the date of pre‐treatment lost‐to‐follow‐up or death. Therefore, we used an estimated date, which was 7 days from the date of registration, based on the average time for treatment initiation in the programme. **Period refers to time before and after the implementation of the person‐centred model of care. In a sensitivity analysis, for which three patients previously exposed to second‐line drugs were excluded from the historical cohort, treatment success was 75.2% (82/109) for the historical cohort, thus similar to 74.1% (83/112) success shown in the primary analysis

At the end of the study period, amongst participants who started the treatment before implementation of the person‐centred care all participants had their outcome defined: 83 (74.1%) achieved treatment success, 13 (11.6%) died, 12 (10.7%) were lost‐to‐follow‐up and 4 (3.6%) were transferred out. Amongst the participants who started the treatment after the implementation of person‐centred model of care 38 (46.3%) participants on short oral regimen had treatment outcomes defined. Treatment success was achieved in 34 (89.5%) participants, 3 died (7.9%) due to reasons not related to TB disease or treatment, and 1 (2.6%) experienced treatment failure due to adverse‐event‐related treatment modifications. The sensitivity analysis, for which three patients were excluded from the historical cohort, showed similar results as the primary analysis (Figure 4, legend).

We compared attrition before with attrition after the adapted care model and observed that pre‐treatment attrition was 23.3% (*n* = 34/146), whilst under the adapted care model it was 5.5% (5/90). Similarly, attrition on treatment was 22.3% (25/112) before the adapted care model, whilst none of the participants were lost‐to‐follow‐up during the implementation of the adapted model and three (3.7%) participants died. Survival after registration into RR‐TB programme significantly improved in the implemented model of care, including SAT and short oral regimen (Figure 4).

## DISCUSSION

In our study, retention in care improved since implementing short oral RR‐TB treatment regimens provided through SAT in war‐torn Kandahar, Afghanistan. We speculate that the acceptability of the care model increased as only a minority of the study population was not retained in care pre‐treatment and no‐one was lost‐to‐follow‐up during treatment, even when the odds of originating from outside of Kandahar province were two times higher in the cohort that benefitted of enhanced person‐centred care. Cultural barriers and levels of insecurity were similar for both study periods. Due to a national lockdown because of the coronavirus pandemic access to care was even more compromised in the period with enhanced person‐centred care. Before SAT and short oral RR‐TB treatments were available, and patients could choose between a long regimen or a short injectable‐containing regimen, a quarter of people with RR‐TB in our programme rejected starting treatment as they were unable to comply with frequent clinic visits and/or long follow‐up periods. Furthermore, almost a quarter of the cohort was not retained in care during their treatment, because they died or were lost‐to‐follow‐up. We attribute improved retention in care to the improved person‐centredness of the adapted care model.

Participants registered in the programme before and after the model of care was implemented had similar demographical and clinical characteristics, including most women. However, there were more children old 15 years or younger and more frequently participants were diagnosed through contact tracing in the period after the model of care was implemented, reflecting our intensified contact tracing activity. Contact tracing has been recognised as a component of a TB programme that maximises early detection and treatment initiation [13]. In our setting, where access to care is extremely challenging, especially for children and women, it was important to use a family approach for a spectrum of TB services provided through the person‐centred model of care. This led to improved diagnosis amongst the vulnerable populations, which are easily missed with the current ‘one‐size‐fits‐all’ models of TB care.

WHO recommends shared decision‐making regarding treatment [5]. Qualitative research findings from South Africa show that people with RR‐TB prefer shorter and injectable‐free regimens and a manageable pill burden, if these regimens also result in a high cure rate [14]. However, the power balance between health care workers (HCWs) and patients remains unclear. Studies from South Africa and Uzbekistan report that the concept of shared decision‐making was unfamiliar to most people with RR‐TB and many chose to defer treatment choice to the HCW, perceived as being knowledgeable and experienced, thus best placed to take such decisions [15]. In Kandahar, levels of patient participation in decision‐making and treatment ownership, and how this affected the patient's motivation to adhere to treatment, needs to be explored through further qualitative studies.

DOT has been used as an intervention to improve patient adherence and its modalities may be adapted, depending on who is providing it and where [16, 17]. Synthesised evidence used to inform the 2018 WHO guidelines reported that person‐centred DOT enhances adherence and improves outcomes compared to unsupervised SAT [16]. People in Afghanistan experience multiple barriers to access health care. Moreover, community‐based programmes may not be secure for HCWs [10]. Most of our cohort originated outside Kandahar, making DOT impossible to implement in our setting. Therefore, we opted for strengthening health education and self‐efficacy, as we offered SAT from 2019 onwards. Our findings are similar to those reported in South Africa, where introducing SAT during the continuation phase of RR‐TB treatment did not affect definitive treatment outcomes [18]. In their context, SAT allowed people to integrate RR‐TB care into their daily life, which reduced the social and economic burden of RR‐TB treatment [19]. Our participants had an option to choose between DOT and SAT and this informed choice may have a positive impact on retention in care. Similar experiences recently documented by Lim et al. reported that patient choice improved self‐efficacy and intention to complete preventative TB treatment in their study in Uganda [20].

Most of our participants came from outside Kandahar. With our model of care, we aimed to reduce the period that patients were separated from their family and home and the need for travel in an insecure environment. However, decentralised RR‐TB care would have brought care closer to their homes and would have had fewer social and economic consequences [21, 22]. Interventions to mitigate economic loss should be integrated into any RR‐TB control strategy. To strengthen and differentiate interventions targeting psychosocial needs and reducing economic burden amongst people with RR‐TB, we need to better understand the role of TB disease and TB treatment in mental and social health and quality of life in this setting.

Our study was not without limitations. The results describe the effect of programmatic modifications over time on TB care outcomes. However, as multiple interventions were implemented at the same time it is difficult to conclude how much the change of treatment regimens or the adaptation of the care model each contributed to the effects observed. The exclusion of patients previously exposed to second‐line drugs from the cohort that benefitted enhanced patient‐centred care could have introduced selection bias, and partially explain better outcomes observed in this cohort. However, a sensitivity analysis showed similar findings when the same exclusion criteria were applied to both cohorts. Furthermore, we acknowledge that cohort that benefited from person‐centred care received care from a more experienced RR‐TB programme. This potential source of residual confounding could not be adjusted for. Bacteriological confirmation of disease was less frequent in the period after the model of care with enhanced person‐centredness was implemented, because a higher proportion of participants were children and because of challenges with sputum sample transport. Lastly, at this stage, we were only able to describe the model of care and estimate its effect on retention in care. Additional patients will need to be enrolled to estimate the effect of the model of care more precisely on less frequent outcomes such as treatment failure. Moreover, whether those who completed will remain relapse‐free can only be assessed through post‐treatment follow‐up. Future, qualitative studies should be conducted to understand whether SAT for all‐oral RR‐TB treatment enhanced ownership, self‐efficacy and motivation for treatment adherence.

As our study findings represent the Kandahar context, results might not be transferable to other settings in the country or beyond. Nevertheless, we believe that our approach, whereby consecutive evaluations informed adaptations to care delivery, may inspire others involved in RR‐TB care.

## CONCLUSION

People living with RR‐TB in Afghanistan are affected by multiple challenges that go beyond TB disease. Short oral treatment regimens for RR‐TB combined with person‐centred care can be used as tools to scale up access to care and improve treatment outcomes.
